# Stress and law enforcers: testing the relationship between law enforcement work stressors and health-related issues

**DOI:** 10.1080/21642850.2013.878657

**Published:** 2014-01-22

**Authors:** Avdi S. Avdija

**Affiliations:** ^a^ Department of Criminology and Criminal Justice, Indiana State University, Terre Haute, IN, USA

**Keywords:** law enforcement, stress, health-related issues, high blood pressure, back pain, alcohol consumption, smoking

## Abstract

This study examines the relationship between law enforcement work-related stressors and health issues. Specifically, this study attempts to determine the effects of stress-producing factors (e.g. vigorous activities at work, shift change, perceived danger associated with police work, etc.) on physiological health-related issues (e.g. the number of reported health issues, high blood pressure, back pain, and headaches) and psychosocial behavior problems (e.g. alcohol consumption and cigarette smoking) among police officers. The analyses are based on a total 1632 law enforcement officers, who represent 51 police agencies in the three major cities, New York City, Dallas Texas, and Minneapolis, USA. The research findings that emerged from this study show that the number of days in vigorous activities and perceived physiological demands have the strongest influence on the number of health-related issues. Working without a partner and frequent shift changes had the strongest influence on alcohol consumption by police officers.

## Introduction

It is no surprise that the law enforcement profession is a stressful job. Officers are repeatedly asked to place their lives in danger to maintain safe neighborhoods. They are exposed to different scenarios that require mental and physical abilities to perform duties efficiently and effectively. The public is aware that law enforcement officers deal with dangerous criminals, and that they must be constantly ready for action each day brings on the job. What the public is less aware of is the amount of stress that comes from daily police-related activities. Although stress is a fact of life, excessive exposure to stress on the job affects officers' well-being both psychologically and physiologically.

### Work-related stressors and physiological problems

There are many different types of law enforcement stressors. Typically, they are classified into four main groups, namely organizational stressors, operational stressors, external stressors, and personal stressors (Dempsey & Forst, 2013). Among the most serious stressors are operational stressors, e.g. experiencing tragedy in the line of duty (Violanti & Aron, 1994). The second group of equally serious stressors are organizational stressors, e.g. shift change, quasi-military style, lack of departmental support for officers when in need, etc. (Shane, 2010; Violanti & Aron, 1994). Generally, work-related stressors, regardless of their type, are known to cause a negative effect on employees' health (Gerber, Hartmann, Brand, Holsboer-Trachsler, & Puhse, 2010; Kawakami & Haratani, 1999; Lundahl, Nelson, Van-Dyk, & West, 2013). Research shows that stress-producing work-related factors can cause physiological problems such as heart attacks, joint pains, headaches, high blood pressure, backaches, diabetes, as well as paralytic stroke (Chen, Tan, & Li, 2013; Joseph et al., 2010; Peterson, Chung, & Swan, 2012). Specific to the law enforcement profession, research shows that police officers are more likely to have health-related issues such as cardiovascular problems, back pain, anxiety problems, depression, and sleep disorders compared to the general public (Gershon, Lin, & Li, 2002; Saha, Sahu, & Paul, 2010; Yoo, 2012). The nature of police work puts police officers at higher risk for health-related problems. Many of the police officers feel that they work under a great amount of pressure and their job is demanding and uncertain, which further contributes negatively to health-related issues. Along these lines, research shows that officers on alternating work schedules suffer greatly from health-related issues (e.g. metabolic syndrome, which includes excess body fat, high blood pressure, high cholesterol, etc.) because they are more fatigued at work and at home (Violanti et al., 2009). Officers transitioning from day shift to night shift are also likely to experience insomnia (Vila & Kenney, 2002). Insomnia is a form of sleeplessness which leads to work fatigue. Work fatigue, on the other hand, is a manifested symptom of stress (Fulle, Pietrangelo, Mancinelli, Saggini, & Fano, 2008) and is detrimental to an officer's health.

### Work-related stressors and psychosocial behavior

In addition to physiological effects, work-related stressors have a negative effect on psychosocial behavior. Research shows that stress increases the likelihood of alcohol consumption (Brady & Pharm, 1999; Keyes, Hatzenbuehler, & Hasin, 2011; Siegrist & Rodel, 2006) and tobacco use among police officers (Smith, Devine, Leggat, & Ishitake, 2005). Many officers turn to drinking at the end of their shift as a way of coping with their stress. Moreover, when officers get together, the conversation is usually work-related. Thus, the officers revisit the same stressful work issues (e.g. exposure to violence at work, exposure to people in pain or distress, etc.) that they were hoping the alcohol would take away (Leino, Eskelinen, Summala, & Virtanen, 2011). This is typically the case with smoking as well. Many officers smoke as a way of coping with their stress. Smoking as a way of coping with stress is an issue for the general population as well (Battig, 1993; Khurshid & Ansari, 2012). A change in an officer's work schedule has also been shown to increase an officer's smoking rate (Smith et al., 2005) in addition to other physiological health issues. Overall, the review of existing literature shows that work-related stressors indeed have a significant impact on officers’ health, both physiologically and psychologically.

## The present study

The law enforcement profession is a unique career. It provides officers with exciting new challenges every day and the job can be very rewarding, while at the same time officers are exposed to the worst of human behavior. The police profession has undergone changes through the years, but what remains the same is the stress the job places on law enforcement officers, which may have a negative impact on officers' health, physically and emotionally. This study examines the relationship between law enforcement work-related stressors and health issues. Specifically, this study attempts to determine the effects of a selected number of work-related police stressors (e.g. vigorous activities at work, shift change, perceived danger associated with police work, etc.) on physiological health-related issues (e.g. the number of reported health issues, high blood pressure, back pain, and headaches) and psychosocial behavior problems (e.g. alcohol consumption and cigarette smoking) among police officers.

## Methodology

### Dependent variables

The dependent variables in this study are the *number of health-related problems*, *high blood pressure*, *work-related back pain*, *headaches, alcohol abuse,* and *smoking*. Three dependent variables were measured at the interval level of measurement. The number of health-related issues was measured by the total number of self-reported health-related problems by the police officers who were surveyed. Alcohol abuse was a categorical variable and it was measured in terms of the number of drinks officers reported consuming during the week (from “none” *coded 0*, to 14+ drinks per week *coded 4*). Cigarette smoking was also a categorical variable. There were four categories for smoking. They were as follows: those who did not smoke at all, *coded 0*, those who smoked 10 or fewer cigarettes per week, *coded 1*; those who smoked 11–20 cigarettes per week, *coded 2*; those who smoked 21–30 cigarettes per week, *coded 3*, and those who smoked 31–40 cigarettes per week, *coded 4*. Because of the interval level of measurement of the first three dependent variables, the ordinary least squares (OLS) multiple regression models (Table 1) were used to test the effect of work-related stressors on the number of health-related issues, alcohol abuse, and smoking among police officers.

**Table 1.** Regression analysis: predicting the number of health-related problems, alcohol abuse, and smoking.

	No. of health problems		Alcohol abuse		Smoking	
*Variables*	*β*	*t*	*β*	*t*	*β*	*t*
(Constant)	–	3.441**	–	3.308***	–	1.633
Age	−.078	−1.567*	−.038	−.719	.122	2.372*
Gender (male)	−.064	−2.544**	.099	3.760***	−.049	−1.883*
Race (white)	.012	0.468	.115	4.300***	.013	.511
Marital status (married)	−.019	−0.655	−.088	−2.869***	−.053	−1.732*
Having children	.005	0.165	−.034	−1.046	.027	.831
Level of education	−.040	−1.548	−.039	−1.452	−.178	−6.677***
Number of years in law enforc.	.090	1.739*	.093	1.702*	.023	.432
Rank within department	.016	0.559	.023	.755	−.012	−.387
Working with partner	−.029	−1.143	.061	2.268*	.016	.610
Frequency of shift change	.040	1.598	.076	2.942***	.015	.606
Days/weeks in vigorous activit.	−.182	−7.305***	.012	.442	−.115	−4.488***
Physiological demands	.250	9.365***	−.001	−.052	−.011	−.417
Danger associated W/job	.065	2.416***	−.028	−1.012	.033	1.178
Used counseling	−.014	−0.564	−.004	−.139	.017	.650
Stigma assoc. W/counseling	.041	1.641	.020	.788	.032	1.235
	No. of health problems		Alcohol abuse		Smoking	
*R*^2^	.128		.046		.073	
*F*-value	*F*(15, 1491) = 14.354		*F*(15, 1491) = 4.781		*F*(15, 1490) = 7.733	
*p*-Value	*p* < .001		*p* < .001		*p* < .001	

*p < .05; **p < .01; ***p < .001

The other three outcome variables included in this study were self-reported high blood pressure by the police officers, measured dichotomously with binary (*yes/no*) response categories, work-related back pain also measured dichotomously with *yes/no* categories, and self-reported headaches by police officers, also measured dichotomously with *yes/no* categories. Given the dichotomous nature of these three dependent variables in this study, the logistic regression models (Tables 2–4) were deemed suitable to test the effects of work-related stressors on the likelihood of police officers reporting high blood pressure, back pain, and headache.

**Table 2.** Logistic regression: predicting high blood pressure among police officers.

Variables in the model	*B*	SE	Wald	Sig.	Exp(*B*)	95% CI for EXP(*B*)	
Age	0.038	0.022	2.884	.089	1.039	0.994	1.086
Gender (male)	1.815	0.608	8.900	.003	6.139	1.864	20.227
Race (white)	−0.636	0.291	4.768	.029	0.530	0.299	0.937
Marital status (married)	−0.116	0.267	0.187	.665	0.891	0.528	1.504
Having children	−0.137	0.284	0.231	.631	0.872	0.500	1.523
Level of education	0.024	0.081	0.088	.767	1.024	0.874	1.201
Number of years in law enforc.	0.062	0.025	6.240	.012	1.064	1.014	1.118
Rank within department	−0.123	0.095	1.677	.195	0.884	0.735	1.065
Working with partner	−0.201	0.222	0.822	.365	0.818	0.529	1.263
Frequency of shift change	−0.150	0.065	5.299	.021	0.861	0.748	0.978
Days/weeks in vigorous activities	−0.215	0.055	15.078	.000	0.807	0.724	0.899
Physiological demands	0.170	0.096	3.126	.077	1.185	0.982	1.431
Danger associated W/job	−0.008	0.095	0.007	.935	0.992	0.823	1.196
Used counseling	0.042	0.313	0.018	.894	1.043	0.564	1.927
Stigma assoc. W/counseling	0.149	0.085	3.040	.081	1.161	0.982	1.372
Constant	−6.648	1.085	37.552	.000	0.001	–	–
(C & S) *R*^2^ = .079 (*N*) *R*^2^ = .175; correct classification of cases 91%

**Table 3.** Logistic regression: predicting work-related back pain among police officers.

Variables in the model	*B*	SE	Wald	Sig.	Exp(*B*)	95% CI for EXP(*B*)	
Age	−0.020	.015	1.752	.186	0.981	0.952	1.010
Gender (male)	0.185	.194	0.902	.342	1.203	0.822	1.761
Race (white)	0.246	.190	1.677	.195	1.279	0.881	1.857
Marital status (married)	−0.159	.162	0.973	.324	0.853	0.621	1.170
Having children	0.097	.166	0.342	.559	1.102	0.796	1.525
Level of education	−0.060	.052	1.310	.252	0.942	0.851	1.043
Number of years in law enforc.	0.027	.016	2.724	.099	1.027	0.995	1.060
Rank within department	−0.098	.066	2.189	.139	0.907	0.796	1.032
Working with partner	−0.382	.132	8.376	.004	0.683	0.527	0.884
Frequency of shift change	0.004	.037	0.013	.909	1.004	0.935	1.079
Days/weeks in vigorous activities	−0.104	.034	9.670	.002	0.901	0.843	0.962
Physiological demands	0.246	.061	16.022	.000	1.279	1.134	1.442
Danger associated W/job	0.037	.059	0.389	.533	1.037	0.924	1.164
Used counseling	−0.106	.226	0.219	.640	0.899	0.577	1.402
Stigma assoc. W/counseling	0.035	.052	0.450	.503	1.036	0.935	1.147
Constant	−1.004	.606	2.742	.098	0.366	–	–
(C & S) *R*^2^ = .036 (*N*) *R*^2^ = .052; Correct classification of cases 73%

**Table 4.** Logistic regression: predicting work-related headaches.

Variables in the model	*B*	SE	Wald	Sig.	Exp(*B*)	95% CI for EXP(*B*)	
Age	−0.030	.015	3.919	.048	0.971	0.943	1.000
Gender (male)	−0.760	.176	18.563	.000	0.467	0.331	0.661
Race (white)	0.020	.180	0.012	.913	1.020	0.716	1.452
Marital status (married)	0.114	.163	0.492	.483	1.121	0.815	1.542
Having children	0.139	.165	0.713	.398	1.149	0.832	1.587
Level of education	0.041	.053	0.593	.441	1.041	0.939	1.155
Number of years in law enforc.	0.007	.016	0.207	.649	1.007	0.976	1.040
Rank within department	−0.086	.069	1.559	.212	0.918	0.802	1.050
Working with partner	−0.068	.129	0.276	.600	0.934	0.725	1.204
Frequency of shift change	0.055	.036	2.434	.119	1.057	0.986	1.133
Days/weeks in vigorous activit.	−0.208	.035	35.378	.000	0.812	0.758	0.870
Physiological demands	0.338	.062	29.410	.000	1.402	1.241	1.584
Danger associated W/job	0.155	.059	6.992	.008	1.168	1.041	1.311
Used counseling	−0.404	.241	2.809	.094	0.668	0.416	1.071
Stigma assoc. W/counseling	0.088	.053	2.768	.096	1.092	0.984	1.212
Constant	0.025	.608	0.002	.967	1.026	–	–
(C & S) *R*^2^ = .087 (*N*) *R*^2^ = .125; correct classification of cases 71.9%							

### Independent variables

This study uses nine independent variables (work-related stressors) and six control variables (mainly demographic characteristics of officers), to test their effects on health-related issues. The nine primary independent variables in this study are treated as identifiers of or work-related stressors that are assumed to have a negative impact on police officers' health. Among the main predictors are officer's rank within the police department, the number of years in law enforcement service, frequency of shift change, working with partner (*coded 1* for yes) or by himself/herself (*coded 0*), the number of days per week in vigorous activities at work, perceived physiological demands in policing, perceived danger associated with police work, counseling used by officers for stress-related issues, and stigma associated with counseling. The control variables, on the other hand, are age, gender, race, marital status, having children, and level of education. The independent variables in this study were coded as follows: the years of service in law enforcement was measured in years; rank within the department was measured from the rank of police officer (*coded 1*) to the rank of captain (*coded 5*); shift change was measured in terms of the frequency of rotation (no shift change, *coded 0*, every six months, *coded 1*, every three months, *coded 2*, every month, *coded 3*, and twice per month, *coded 4*). The perceived physiological demands in law enforcement work was measured using one 5-point Likert-scale item with response categories from “not at all” *coded 1* to “a lot” *coded 5*. Whether the police used counseling or not was measured dichotomously with *yes/no* categories. The stigma associated with counseling was measured using one 5-point Likert-scale item with response categories from “strongly disagree” *coded 1* to “strongly agree” *coded 5*.

Four of six variables that measured demographic characteristics of police officers were dichotomously measured. Gender was coded females *0*, males *coded 1*. Race was also a dichotomous variable, *coded 0* for non-whites, *coded 1* for whites. Marital status was measured with two binary response categories (single, *coded 1*, and married, *coded 2*). Having children was *coded 1* for yes, otherwise *0*. The level of education was a categorical variable with six categories, ranging from high-school education, *coded 1*, to graduate degree, *coded 6*. Age was measured in years as self-reported by the police officers.

## The data source and participants

This study analyzes the data that were released to the public in 2005 by the Interuniversity Consortium for Political and Social Research (ICPSR). The data were originally gathered by Delprino, Robert, Karen O'Quin, and Cheryl Kennedy from three major US cities, New York City, Dallas, and Minneapolis, in 1995 as part of a larger study on work-related issues and their effects on police families. From this data set, I have attempted to identify relevant police work-related stressors and their effect on selected health-related issues. The analyses are based on a total of 1632 usable cases, which represent 51 police agencies in the three major cities mentioned above. The descriptive statistics show that 1421 (87.1%) of the sample were male officers and 208 (12.7%) were female officers. The average age was 37 (SD = 8.42), and they ranged in age from 22 to 64 years. In terms of race, 1382 (84.7%) were whites and 242 (14.8%) non-whites. About three-fourths of them (72.3%) were married and 27.3% were single. The average years of service in law enforcement was 12.86 years (SD = 7.95). In terms of the ranks within the department, 964 (59.1%) of them were police officers, 222 (13.6%) were detectives, 282 (17.3%) were sergeants, 90 (5.5%) lieutenants, and 35 (2.4%) captains.

## Results

The first step in the analyses was to check if there were any issues that would violate the multicollinearity assumption with the data set. First, I computed the Spearman correlation matrix to see the degree of correlation between the predictors, and then I checked the variance inflation factor (VIF) values. The correlations between predictors ranged from *r_s_* = .010 to *r_s_* = .530. Multicollinearity becomes an issue when independent variables are highly correlated. Correlations that present a problem with multicollinearity are those that reach *r* = .9 or above (Pallant, 2011). In this study, all correlations between variables were within the normal range. In addition to the correlation matrix, the VIF values were examined as well. The VIF values ranged from 1.051 to 4.225. These scores were well below the cut-off value of 10. The VIF values below 10 indicate that the data do not suffer from multicollinearity (Pallant, 2011). In light of these two statistical analyses, it is safe to conclude that the data did not have any issues with the multicollinearity in this study. The next step of analyses was OLS regression, predicting the influence of work-related stressors on the number of health-related problems, alcohol abuse, and cigarette smoking by police officers.

The multiple regression analyses are presented in Table 1. The analyses in Table 1 show that all three models were statistically significant. After controlling for the demographic characteristics of police officers (e.g. age, gender, race, marital status, having children, and level of education), four variables were statistically significant in predicting the number of health-related problems, namely, the number of years in law enforcement (*β* = .090, *p* < .05), the number of days per week in vigorous activities (*β* = −.182, *p* < .001), perceived physiological demands in police work (*β* = .250, *p* < .001), and perceived danger associated with the police work (*β* = .065, *p* < .001). Additionally, among the control variables, age and gender had a statistically significant effect on the number of health-related problems. It should be noted that the perceived physiological demands had the largest impact (*β* = .250) on the number of health-related issues. Thus, for every standard deviation unit increase in the perceived physiological demands by police officers, there was a 0.250 standard deviation unit increase in the number of health-related issues, as reported by the police officers. The number of days per week in vigorous activities had a negative impact on the number of health-related issues. This means, an increase in the number of days per week in vigorous activities is manifested with fewer health-related problems.

Regarding alcohol consumption (or alcohol abuse), the data in Table 1 show that the frequency of shift change (*β* = .076, *p* < .001), working with a partner (*β* = .061, *p* < .05), and the number of years in law enforcement service (*β* = .093, *p* < .05) had a statistically significant effect on alcohol consumption among police officers. Gender, race, and marital status (being single) also had a significant impact on alcohol consumption. This suggests that being a white male, single, who works with a partner, and is exposed to frequent shift changes, for a longer period of time (years of service) has a higher probability of consuming alcohol compared to other corresponding officers with opposite characteristics.

The third outcome variable that was tested in this study was the number of cigarettes smoked by officers per week. The data in Table 1 show that five variables made a statistically significant contribution in predicting smoking among police officers. Among the non-demographic variables, only the number of days per week in vigorous activities was statistically significant in predicting smoking (*β* = −.115, *p* < .001). The level of education, marital status, and gender had a negative impact on smoking. Age had a positive impact on smoking (*β* = .122, *p* < .05). This means that being a married female officer with a higher level of education reduces the probability of cigarette smoking. On the other hand, the data show that as age increases, the probability of smoking increases also, holding all other variables constant.

Three additional outcome variables that were tested in this study were dichotomous in nature. Thus, because of their binary response categories, logistic regression models were used to predict the likelihood of police officers reporting high blood pressure, work-related back pain, and headaches due to a selected number of law enforcement work-related stressors. The data are presented in Tables 2–4. The analyses show that all three logistic models (Tables 2–4) were statistically significant, indicating that the models were able to distinguish between those police officers who reported problems with high blood pressure *χ* ^2^ (15, *N* = 1492) = 123.246, *p* < .001, work-related back pain *χ* ^2^ (15, *N* = 1492) = 54.019, *p *< .001, and headaches *χ* ^2^ (15, *N* = 1492) = 135.927, *p *< .001 and those who did not report such problems.

The analyses in Table 2 show that there were four variables that made a statistically significant contribution in the model. Controlling for the demographic variables, frequency of shift change and the number of days per week in vigorous activities were among the factors that had a positive effect on the high blood pressure, as reported by the police officers. The odds ratio for the frequency of shift change was 0.861, indicating that officers who reported an increased frequency in shift change were 13.9% less likely to report problems with high blood pressure compared to those who did not. This means, the higher the frequency of shift change, the less likelihood of having problems with high blood pressure. The odds ratio for the number of days per week in vigorous activities was 0.807, indicating that for each additional day's involvement in vigorous activities per week, there was a decrease in the likelihood of reported having problems with high blood pressure by 19.3%. Additionally, gender, race, and the number of years in law enforcement service had a significant impact on high blood pressure. It is noteworthy that gender recorded an odds ratio of 6.139, indicating that male officers were six times more likely to report problems with high blood pressure compared to female officers.

The same variables were used to predict work-related back pain among police officers. The data in Table 3 show that only three variables made a statistically significant contribution to the model, namely, perceived physiological demands, the number of days per week in vigorous activities, and working with partner. The strongest impact on work-related back pain had the perceived physiological demands, which recorded an odds ratio of 1.279. This indicates that the odds of reporting work-related back pain were 1.2 times higher for the police officers who perceived police work as physiologically demanding compared to those who did not, holding all other variables constant. Contrary to this, the number of days per week in vigorous activities contributed to a decrease in the likelihood of reporting work-related back pain. The odds ratio for the number of days per week in vigorous activities was 0.901, indicating that for each additional day in vigorous activities, the odds of reporting back pain decreased by a factor of 0.901.

The third logistic model (Table 4) tested the effects of the same 15 variables on work-related headaches. Only four variables made a statistically significant contribution to the work-related headaches model. The strongest influence on work-related headaches had the perceived physiological demands in the police work by police officers, recording an odds ratio of 1.404. This indicates that the odds of a police officer answering *Yes*, they have frequent headaches, was 1.4 times higher for those officers who perceived the police work as physiologically demanding compared to those do did not. Perceived danger associated with the job recorded an odds ratio of 1.168. This means that the odds of officers who perceived danger associated with the police work, reporting having work-related headaches, increased by a factor of 1.168 compared to those who did not; holding all other variables constant. Contrary to this, the number of days per week in vigorous activities recorded an odds ratio of 0.812, indicating that for each additional day in vigorous activities, the odds of reporting headaches decreased by 18.8%. Moreover, the data in Table 4 show that being a male officer decreased the likelihood of having problems with headaches by a factor of 0.467 compared to female officers.

## Discussion

Using a 15-variable model, the purpose of this study was to examine the relationship between law enforcement work-related stressors and health issues among police officers. The outcome variables in this study were the number of health-related issues as reported by the police officers, alcohol abuse, cigarette smoking, high blood pressure, work-related back pain, and work-related headaches. The data were analyzed using OLS regression models, and for the dichotomous outcome variables, logistic regression models were computed. All six models presented in the results section were statistically significant, indicating that at least some of the variables in the models made a statistically significant contribution in explaining the effect of a selected number of work stressors on specific work-related health issues that were included as outcome variables in this study. The unique contribution of this research study to the existing literature relates to the inclusion of perceived physiological demands about police work and its effects on health-related issues, including specific health problems such as high blood pressure, back pain, and headaches.

### Number of health-related issues, alcohol abuse, and smoking

Overall, the research findings of this study indicate that police officers who perceived the police work as physiologically demanding reported a higher number of health-related issues. Thus, an increased score in perceived physiological demands about police work, measured in standardized unites, was manifested with an increase in the number of reported health-related issues. Surprisingly, this study shows that age was negatively associated with the reported number of work-related issues, including alcohol abuse. Older police officers reported fewer health-related issues and were less likely to consume alcohol. Also, the number of days per week in vigorous activities had a negative impact on the number of health-related issues and cigarette smoking by police officers. This means, an increase in the number of days per week in vigorous activities is manifested with fewer health-related problems and a reduced probability of smoking cigarettes, an unexpected finding. Furthermore, the findings of this research study indicate that being a white male, single, who works with a partner, and is exposed to frequent shift changes, for a longer period of time, has a higher probability of consuming alcohol compared to other corresponding officers with opposite characteristics. The results of this study are consistent with prior research, which indicates that police officers who are frequently exposed to work-related risk factors/stressors are more likely to consume alcohol (Gershon et al., 2002). On the other hand, this research indicates that being married, female officers with a higher level of education reduces the probability of cigarette smoking. Contrary to gender and marital status effect, this study shows that as age increases, the probability of smoking increases also; a finding that concurs with prior research (Center for Disease Control and Prevention, 2011).

### High blood pressure

The findings of this study show that officers who have been longer in the law enforcement profession have a higher likelihood of developing high blood pressure, as reported by officers. Additionally, male police officers were six times more likely to answer *Yes*, they have developed high blood pressure, compared to female police officers. The frequency of shift change and the number of days per week in vigorous activities were negatively associated with reported high blood pressure. This means, the higher the frequency of shift change, the less likelihood of having problems with high blood pressure. Also, consistent with prior research regarding the effects of physical activities on high blood pressure (see Arrol & Beaglehole, 1992; Kelley, Kelley, & Tran, 2001; Padilla, Wallace, & Park, 2005; Whelton, Chin, Xin, & He, 2002), this study shows that for each additional day's involvement in vigorous activities per week, there was a decrease in the likelihood of having problems with high blood pressure. Overall, a typical profile of an officer who reported having problems with high blood pressure includes being a non-white, male officer, who has been in law enforcement service for long time, less involved in vigorous activities, and works in the same shift without being exposed to frequent shift rotations.

### Back pain & headaches

Police work is unique in terms of physical activities. Typically, when there are no emergencies to respond to, police officers spend hours on routine patrols, which means driving for longer periods of time. Research shows that individuals who spend more hours driving are more likely to develop back pain (Guo, 2002; Krause et al., 1997). Research also shows that physiological work demands have a negative effect on the neuromuscular lower back (Gershon et al., 2002; Hui, Ng, Yeung, & Hui-Chan, 2001). Among many police activities, driving is considered a physiologically demanding work-related activity. This study shows that perceived physiological demands, although psychological in nature, had a strong impact on work-related back pain. The odds of reporting work-related back pain were 1.2 times higher for the police officers who perceived police work as physiologically demanding compared to those who did not. Perceived physiological demands had a strong impact on reported work-related headaches also. The odds of a police officer answering *Yes*, they have frequent headaches, was 1.4 times higher for those officers who perceived the police work as physiologically demanding compared to those do did not. Contrary to this, the number of days per week in vigorous activities contributed to a decrease in the likelihood of reporting work-related back pain and headaches. The findings of this study show that for each additional day per week in vigorous activities, the odds of reporting back pain decreased by a factor of 0.901 and for headaches decreased by a factor of 0.812. Moreover, perceived danger associated with the job had a strong effect on the reported number of headaches and total number of health-related issues, as reported by the police officers. Officers who perceived danger associated with the police work were 1.168 times more likely to report they have frequent headaches compared to those who did not. Also, the results of this study show that being a male officer decreased the likelihood of having problems with headaches compared to females.

### Limitations of this study

There are a few research limitations of this study that are noteworthy. First, the 15-variable model that was used in this study does not include an exhaustive list of work-related stressors in the law enforcement profession. The number of variables was limited to 15 because of the actual limitations in the data set. Second, the survey results in the data are self-reported. In other words, the information that was reported by the police officers may be an underestimate or overestimate of the actual number of health-related problems. A more accurate estimation of the total number of and the types of health-related problems among police officers would be to crosscheck them with the actual medical history of the participants, which was not available or accessible. Third, due to the limitations in the data set, this study is limited to considering only the alcohol consumption and cigarette smoking as relevant aspects of the psychosocial behavior problems met by the police officers. Future research can address the violence and arbitrary exercise of power, in addition to alcohol consumption and cigarette smoking among the psychosocial behavior problems that were included in this study.
